# Circulating serum miR-362-3p and miR-6721-5p as potential biomarkers for classification patients with adult-type diffuse glioma

**DOI:** 10.3389/fmolb.2024.1368372

**Published:** 2024-02-22

**Authors:** Magdalena Niemira, Agnieszka Bielska, Karolina Chwialkowska, Justyna Raczkowska, Anna Skwarska, Anna Erol, Anna Zeller, Gabriela Sokolowska, Damian Toczydlowski, Iwona Sidorkiewicz, Zenon Mariak, Joanna Reszec, Tomasz Lyson, Marcin Moniuszko, Adam Kretowski

**Affiliations:** ^1^ Clinical Research Centre, Medical University of Bialystok, Bialystok, Poland; ^2^ Centre for Bioinformatics and Data Analysis, Medical University of Bialystok, Bialystok, Poland; ^3^ Albert Einstein College of Medicine, Cancer Center, Bronx, NY, United States; ^4^ Department of Neurosurgery, Medical University of Bialystok, Bialystok, Poland; ^5^ Department of Medical Pathology, Medical University of Bialystok, Bialystok, Poland; ^6^ Centre of Regenerative Medicine, Medical University of Bialystok, Bialystok, Poland

**Keywords:** miRNA, serum, non-invasive biomarkers, gliomas, diagnostic model

## Abstract

According to the fifth edition of the WHO Classification of Tumours of the Central Nervous System (CNS) published in 2021, grade 4 gliomas classification includes IDH-mutant astrocytomas and wild-type IDH glioblastomas. Unfortunately, despite precision oncology development, the prognosis for patients with grade 4 glioma remains poor, indicating an urgent need for better diagnostic and therapeutic strategies. Circulating miRNAs besides being important regulators of cancer development could serve as promising diagnostic biomarkers for patients with grade 4 glioma. Here, we propose a two-miRNA miR-362-3p and miR-6721-5p screening signature for serum for non-invasive classification of identified glioma cases into the highest-grade 4 and lower-grade gliomas. A total of 102 samples were included in this study, comprising 78 grade 4 glioma cases and 24 grade 2–3 glioma subjects. Using the NanoString platform, seven miRNAs were identified as differentially expressed (DE), which was subsequently confirmed via RT-qPCR analysis. Next, numerous combinations of DE miRNAs were employed to develop classification models. The dual panel of miR-362-3p and miR-6721-5p displayed the highest diagnostic value to differentiate grade 4 patients and lower grade cases with an AUC of 0.867. Additionally, this signature also had a high AUC = 0.854 in the verification cohorts by RT-qPCR and an AUC = 0.842 using external data from the GEO public database. The functional annotation analyses of predicted DE miRNA target genes showed their primary involvement in the STAT3 and HIF-1 signalling pathways and the signalling pathway of pluripotency of stem cells and glioblastoma-related pathways. For additional exploration of miRNA expression patterns correlated with glioma, we performed the Weighted Gene-Co Expression Network Analysis (WGCNA). We showed that the modules most associated with glioma grade contained as many as six DE miRNAs. In conclusion, this study presents the first evidence of serum miRNA expression profiling in adult-type diffuse glioma using a classification based on the WHO 2021 guidelines. We expect that the discovered dual miR-362-3p and miR-6721-5p signatures have the potential to be utilised for grading gliomas in clinical applications.

## 1 Introduction

Adult-type diffuse gliomas are the most prevalent tumours, accounting for approximately 80% of all central nervous system malignant tumours (Siegel et al., 2023). They are the leading cause of significant morbidity and mortality due to high invasiveness, accelerated growth, dismal localisation, and heterogeneity (Louis et al., 2021). Regarding the latest WHO Classification of Central Nervous System (WHO CNS5) adult-type diffuse gliomas, three dominant types can be distinguished: i) oligodendroglioma, with isocitrate dehydrogenase IDH mutations and 1p/19q codeletion; ii) astrocytoma with IDH mutations; iii) and glioblastoma with wild-type IDH (Louis et al., 2021; Osborn et al., 2022). IDH-mutated astrocytoma and wild-type IDH glioblastoma (GBM are now classified as grade 4 cancer. This type of tumour is particularly invasive, destructive and aggressive (McNamara et al., 2022; Yang et al., 2022). Currently, diagnosis of grade 4 gliomas remains limited and is based on neurological testing and neuroimaging (Silantyev et al., 2019). The ultimate glioma grade determination solely relies on a histological examination utilising previous tumour resection. Moreover, biopsies are used, but they can be misleading because of the heterogeneity of the tumour (Weller et al., 2015). Thus, there is an urgent need to identify non-invasive biomarkers that would help to classify patients with different grades of glioma and recognise individuals with the predisposition to its development.

MicroRNAs (miRNAs) are small, non-coding, endogenous single-stranded RNAs with about 17–25 nucleotides in length (Huang, 2017). The formation of miRNA is a complex process that involves multiple steps, beginning in the cell nucleus and ending in the cytoplasm. Genes encoding miRNAs are transcribed by polymerase II into pri-miRNA. The pri-miRNA is processed into pre-miRNA with the involvement of Drosha ribonuclease. The pre-miRNA is then transported from the nucleus to the cytoplasm of the cell. Dicer endonuclease cleaves the pre-miRNA into short miRNA duplexes, which are later unfolded by an unknown helicase. The mature miRNA strand binds to the Ago protein to form a complex (O’Brien et al., 2018; Shang et al., 2023). These molecules play a vital role in regulating post-transcriptional regulation of gene expression by interacting with the 3′ untranslated region (3′UTR) of their target messenger RNA (mRNA). The degree of complementarity between the miRNA sequence and its target mRNA determines the regulatory effect of miRNA. High nucleotide complementarity usually leads to transcript degradation, while partial complementarity inhibits translation (Bhowmick et al., 2018). A single miRNA can target and regulate many genes (Doench and Sharp, 2004). Interestingly, one miRNA molecule can regulate hundreds of genes, but one gene can also be regulated by dozens of miRNAs. Bioinformatics analyses predict that nearly two-thirds of human genes can be regulated by the action of miRNAs (Quillet et al., 2020). MiRNAs are involved in various cellular processes such as development, proliferation, apoptosis, metabolism, differentiation, metastasis, angiogenesis, and tumourigenesis (Tiwari et al., 2017; Vishnoi and Rani, 2017; Bautista-Sánchez et al., 2020).

In recent years, research has emphasised the crucial role miRNA may play as a biomarker for many diseases. Changes in miRNA expression have been linked to the development of many civilization diseases such as cancer, cardiovascular disease, and metabolic disorders. MiRNA expression profiling is believed to be a useful diagnostic tool for early disease detection, disease severity assessment, treatment response monitoring, and personalized therapeutic approaches (Condrat et al., 2020). MiRNA has several features which make it an ideal candidate for this role. Firstly, it can be collected with minimal invasiveness from biofluids that are easily accessible, such as saliva, serum, plasma or urine (Huang, 2017; Lu and Rothenberg, 2018). In those biofluids, miRNAs can be packaged in various extracellular vehicles, including exosomes, microvesicles, and apoptotic bodies, as well as bound to proteins like Ago2 or HDL molecules, which provides higher stability (Nik Mohamed Kamal et al., 2020). Even after repeated freezing and thawing, the stability of miRNA’s structure in biofluids is a significant advantage (Banno et al., 2014). Collecting biofluids from patients through liquid biopsy is a minimally invasive and straightforward process. This makes diagnostics more accessible and less expensive than tissue biopsy, which is significant in terms of brain tumour (Eibl and Schneemann, 2023). In recent years, miRNAs have gained importance as biomarkers for diagnosis, prognosis, and developing therapeutic strategies for breast, prostate, and lung cancers, melanoma, but also cardiovascular and neuronal diseases (Faruq and Vecchione, 2015; Huang, 2017; Wang et al., 2018; Bielska et al., 2021). Dysregulation of miRNA expression is closely associated with the initiation, progression, and metastasis of cancer. The diversity of miRNA-regulated genes implies that these molecules can act as oncomiRs or as suppressors in the context of cancer development (Bautista-Sánchez et al., 2020). OncomiRs can promote tumour development by targeting tumour suppressor (Ts), whereas tumour suppressor miRNAs work by suppressing oncogenes, leading to the inhibition of tumour progression (Banno et al., 2014; Lin and Gregory, 2015).

Recent studies suggest that certain serum miRNAs have the potential to diagnose glioma or distinguish its grade. Most research on miRNA in glioma patients is based on the previous histological classification of gliomas according to the 2016 WHO classification. At that time, low-grade gliomas (LGG) belonged to grade I or II I–II, while high-grade gliomas (HGG) were classified as grades III–IV, including GBM (Louis et al., 2016). Using this classification, Wei et al. (2016) found that the level of miR-125b in serum was significantly reduced in glioma patients compared to healthy controls, and the gradual decrease in miR-125b levels was clearly discernible at the stage rose. Additionally, Regazzo et al. (2016) reported that miR-125b and miR-497 had a diagnostic value in differentiating between GBM and lower-grade glioma cases. Wang et al. (2019) revealed that upregulated cell-free miR-214 in serum was significantly associated with higher tumour grade, absence of isocitrate dehydrogenase, and unmethylated methylguanine methyltransferase promoter.

Further studies shed light on the potential of serum miR-100 as a promising biomarker for GBM diagnosis. The levels of this miRNA, measured by qPCR, were significantly higher in GBM patients than in healthy subjects (Zhang et al., 2019). Zhu et al. (2019) discovered that the expression of miR-193b in serum and tumour tissue was notably higher than in the non-tumour samples. The AUC value for this miRNA exceeded 0.90, indicating its high prognostic potential for glioma. A recent study has demonstrated that miR-582-5p and miR-363 in serum can effectively distinguish GBM patients from healthy individuals with remarkable specificity and sensitivity. The prognostic value of miR-575 in GBM patients was demonstrated by Gray et al. (2022) This miRNA promotes tumour progression and is associated with worse overall survival. Géczi et al. (2021) found that 53 miRNAs were significantly differentially expressed in plasma samples of patients with GBM compared to healthy donors. However, the main limitation of this study was the small sample size, which included only six GBM patients and six healthy controls. In one of the latest studies, Sun et al. (2021) suggested that plasma exosomal miR-2276-5p could potentially serve as a biomarker for patients with glioma. The expression of this specific miRNA was considerably decreased in patients with glioma, which correlated with a reduced survival rate.

Despite numerous studies on potential biomarkers in glioma, this area still requires standardisation, consistency and unification. More knowledge regarding the specific serum miRNA profile in patients with grade 4 of adult-diffuse gliomas classified based on CNS5 WHO standards remains needed. Relatively easy analysis of miRNA expression can significantly support the diagnosis, particularly for patients with highly localised tumours and at increased risk of perioperative mortality. In addition, examining miRNA levels can be done recurrently, which could help to monitor disease progression and response to treatment (Śledzińska et al., 2021).

In the presented study, we are the first to report a serum miRNA-based glioma 4 diagnostic model with high discriminative ability in classifying grade 4 glioma patients from grade 2–3 glioma cases. We also investigated the possible involvement of circulating miRNAs in the highest-grade glioma development by analysing the biological importance of miRNA targets and the functional enrichment profile of their gene sets.

## 2 Materials and methods

### 2.1 Study cohort

In the presented study, serum samples were collected in the Clinical Hospital in Bialystok by Biobank at the Medical University of Bialystok, with high standards of strict biobanking procedures for multi-omics studies (Niklinski et al., 2017; Michalska-Falkowska et al., 2023). The study group consisted of patients qualified for surgical treatment of adult-type diffuse gliomas at different stages of development (WHO, 2021 grade 2–4). All patients were grouped into 24 lower-grade gliomas CNS5 WHO grade 2 and 3 and 78 WHO grade 4 (Table 1). The serum was collected before any treatment of cancer patients was started. The serum samples were collected, centrifuged and stored at −80°C. A group of 102 individuals was qualified for further analysis. The exclusion criteria for cases were patients diagnosed with other types of cancer than an adult-type diffuse glioma, chemotherapy or radiotherapy before serum collection and no cancer history. All participants provided written informed consent and received detailed information on the study and associated risks before enrolment. This study was approved by the Bioethics Committee of the Medical University of Bialystok, Poland (approval numbers: R-I-002/357/2014, R-I-002/600/2019, and APK.002.171.2021) and was performed according to the principles of the Declaration of Helsinki.

**TABLE 1:** Clinicopathological characteristics of the study cohort.

Characteristics	Adult-type diffuse gliomas (%)		
	WHO CNS5 grade 4 *n* = 78	WHO CNS5 grade 2-3 *n* = 24	*P* val
Age at diagnosis (mean ± SD)	58.2 ± 10	48.7 ± 12	0.4
BMI (mean ± SD)	27.2 ± 5.0	26.7 ± 5.6	0.8
Size of tumour [mm] (mean ± SD)	4.4 ± 1.7	3.8 ± 2.1	0.3
Histological diagnosis (WHO 2021)			
Astrocytoma	16 (20.5)	21 (87.5)	
Oligodendroglioma	-	3 (12.5)	
Glioblastoma	62 (79.5)	-	
*Molecular biomarkers*			
IDH1/2-mutant (MT)	16 (20.5)	24 (100)	
IDH1/2-wildtype (WT)	62 (79.5)	-	

### 2.2 Sample size estimation

Based on similar previous experiments and pilot data, we have calculated the minimal number of samples per experimental group (grade 4 or grade 2–3) to detect two-fold differences in relative expression levels between groups at the true positive detection powers of 80% and 90% (Hart et al., 2013). We have used the RNASeqPower R package to apply the statistics data covering obtained real counts and coefficients of variations per group. For the NanoString nCounter miRNA data, we have estimated that to obtain 80% power, we would need nine samples per group, whereas to obtain a high power of 90%, we would need 12 samples. Finally, our groups for NanoString analyses consisted of 78 (grade 4) and 24 (grade 2–3) samples, thus allowing for more than 90% power in any of the comparisons performed.

### 2.3 RNA preparation and miRNA profiling by NanoString

RNA isolation with miRNA fraction serum samples was performed using the miRNeasy Serum/Plasma Advanced Kit (Qiagen, Germany) according to the manufacturer’s instructions. A total of 102 samples were analysed using the nCounter®Analysis System (NanoString Technologies, WA, United States) and the nCounter Human v3 miRNA Panel. Briefly, as input material, 3 ng of isolated miRNA was used. Unique RNA tags were ligated onto the 3′ end of each mature miRNA, followed by an overnight hybridisation (65°C) to nCounter Reporter and Capture probes. After hybridisation, samples were placed into the nCounter Prep Station for sample purification and target/probe complexes immobilisation on the cartridge. For each assay, a high-density scan (555 fields of view) was performed on the nCounter Digital Analyzer (NanoString Technologies, United States) to count individual fluorescent barcodes and quantify target miRNA molecules present in each sample.

### 2.4 qPCR

The seven DE miRNAs and two reference miRNAs were profiled using the miRCURY LNA SYBR Green PCR Kit (Qiagen, Germany). Reference miRNAs with stable expression across all samples, has-miR-103-3p and has-199b-5p, were selected based on the NanoString data using the NormFinder algorithm (Andersen et al., 2004). The miRCURY LNA RT Kit (Qiagen, Germany) was used for the reverse transcription reaction for miRNA assays. Assay IDs are assembled in Supplementary Table S1. Quantitative real-time PCR (RT-qPCR) was performed with miRCURY LNA SYBR^®^ Green PCR Kits (Qiagen, Germany) with specific commercial primers, optimized with LNA technology to enable sensitive and specific miRNA quantification. For *STAT3*, *FOS* and *TLR4* mRNA analysis, we used the Transcription High Fidelity cDNA Synthesis Kit (Roche, Switzerland) and KiCqStart^®^ SYBR^®^ Green qPCR ReadyMix™ (Sigma-Aldrich, Germany). The following primers were used: *STAT3* FW-CTTTGAGACCGAGGTGTATCACC; *STAT3* RV-GGTCAGCATGTTGTACCACAGG; *FOS* FW-GCCTCTCTTACTACCACTCACC; *FOS* RV-AGATGGCAGTGACCGTGGGAAT; *TLR4* FW-CCCTGAGGCATTTAGGCAGCTA; *FOS* RV-AGGTAGAGAGGTGGCTTAGGCT; *GAPDH* FW GTC​TCC​TCT​GAC​TTC​AAC​AGC​G; GAPDH RV-ACCACCCTGTTGCTGTAGCCAA. The temperature profile of the qPCT reaction was as follows: 2 min at 95°C and 45 cycles: 10 s at 95°C and 60 s at 56°C (for miRNA) and 60°C (for mRNA). Amplification was performed using LightCycler 480 (Roche, Switzerland). Subsequently, PCR threshold cycles (Ct) of the tested miRNA/mRNA and reference miRNA/mRNA were determined for the tested samples and the calibrator. The relative expression for each miRNA/mRNA was calculated with PCR efficiency correction (Pfaffl, 2001). Efficiency (E) was calculated from the slopes of the calibration curve according to the equation: E = 10 (−1/slope). Reactions with amplification efficiency below 1.6 were removed. The relative expression ratio of a target miRNA was computed based on its PCR efficiencies (E) and the Ct value difference (Δ) of unknown group samples (test) *versus* the control group (Δ Ct control-test). The relative calculation was based on the MEAN Ct of the experimental group.

### 2.5 Functional enrichment analysis and network construction

The identification of DE miRNA target genes was performed using Ingenuity Pathway Analysis Software (Krämer et al., 2014) (IPA, Qiagen Inc. https://qiagenbioinformatics.com/products/ingenuity-pathway analysis), mirDB database (Chen and Wang, 2020) (https://mirdb.org), and TagetScanHuman 8.0 database (Agarwal et al., 2015) (https://www.targetscan.org/vert_80/). Gene Ontology Biological Process and KEGG Pathway analyses were conducted with the ClusterProfile R package (Yu et al., 2012). IPA was used to perform the core analysis to identify canonical pathways. Over-representation analyses used hypergeometric tests under α = 0.05 (*p*-values corrected with FDR). To construct a PPI network, we used STRING (https://string-db.org/; v.11.5). Genes with a confidence score ≥0.4 were chosen to build a network model visualised by Cytoscape v.3.9.1. Nine topological algorithms in plug-in *cytoHubba* (Chin et al., 2014), consisting of “MCC,” “MNC,” “Degree,” “Bottle Neck,” “EcCentricity,” “Closeness,” “Stress,” and “Radiality” were selected to identify the hub genes in PPI analysis. Analyses of functional interaction networks were based on the HumanNet v3 platform by applying the HumenNet-FN (functional gene network) mode (https://www.inetbio.org/humannet/) (Kim et al., 2022).

### 2.6 WGCNA analysis

The discovery and the analysis of miRNA co-expression modules in the patient’s tumour samples based on NanoString data were performed with Weighted Gene Coexpression Network Analysis (WGCNA) (Langfelder and Horvath, 2008) by application of the WGCNA and CEMiTool R libraries (Russo et al., 2018). Outliers were removed based on standardised connectivity. Pearson correlation was used in the signed network construction with soft threshold selection (R2 > 0.8). Modules were merged based on a high eigengene similarity correlation threshold (0.85). Gene modules to trait relationships were evaluated with Spearman correlation. Within each WGCNA module, an interaction network was analysed using CEMiTool. Interaction networks integrate the miRNA co-expression results with miRNA-target gene annotations based on miRTarBase 8.0 (Huang et al., 2020). Database miRTarBase provides comprehensive information on experimentally validated miRNA-target interactions from various literature data and other databases that have been data mined and manually curated. The top ten most connected genes were identified as network hubs and are indicated with text labels and coloured based on the interaction type (miRNA co-expression pattern–blue; miRNA-target gene interaction–red; miRNA co-expression pattern and miRNA-target gene interaction–yellow). The size of each node is proportional to its degree, as indicated in the plot legend.

### 2.7 Diagnostic model development

The normalised NanoString counts and RT-qPCR data were used for model development. The attributes were selected using Waikato Environment for Knowledge Analysis (WEKA) version 3.8.3. (c) 1999–2018 The University of Waikato, Hamilton, New Zealand. The InfoGainAttributeEval algorithm was used to select the best classifiers. Feature selection via information gain using InfoGainAttributeEval was based on the calculation of decreasing entropy by adding attributes and selection attributes that most strongly reduce entropy. This method was carried out on the testing set using the LOOCV (Wainer and Cawley, 2021; Rafało, 2022). A multivariate logistic regression model was built using repeated (*n* = 3) k-fold-cross-validation (*k* = 10) in R version 3.6.1 [R Core Team (2013) R: A language and Environment for Statistical Computing. R Foundation for Statistical Computing, Vienna. https://www.R-project.org] (Kuhn, 2008). To validate the model, the ROC curve and the AUC were calculated by the pROC package (Robin et al., 2011). The confusion matrix, including information about TP (true positives), TN (true negatives), FP (false positives), and FN (false negatives) has been prepared. The evaluation of model classification has been based on the testing dataset. The choice of miRNAs was evaluated using the independent, external and publicly available dataset GSE112462 from the Gene Expression Omnibus (GEO) database (https://www.ncbi.nlm.nih.gov/geo/query/acc.cgi?acc=GSE112462), which contains miRNA profiles of 28 glioma patients (18 patients with grade 2–3 and 10 patients with grade 4) (Drusco et al., 2018). This external data was generated using NanoString human miRNA panel NS_H_MIR_V3A. A logistic regression model was developed for this data according to the above workflow.

### 2.8 Statistical analysis

Statistical analyses were performed using GraphPad Prism 9 (v.9.3.1) software. The Wilcoxon rank sum test was used to investigate the differences in BMI, age and size of tumours between the grade 4 patients’ group and grade 2–3. Raw miRNA data were analysed using nSolver Software v. 4.0 (NanoString Technologies, WA, United States). Code-set content normalisation was performed relative to the ligation controls for technical variations. Ratios were calculated by specifying the lower-grade samples as a baseline. According to Benjamin-Hochberg, correction for multiple testing was performed with a False Discovery Rate (FDR). The Pearson correlation coefficient (r) was used to estimate the correlation between the identified DE miRNAs and clinical parameters.

## 3 Results

### 3.1 Detection of circulating miRNAs in serum samples from glioma patients before surgery

**FIGURE 1:**
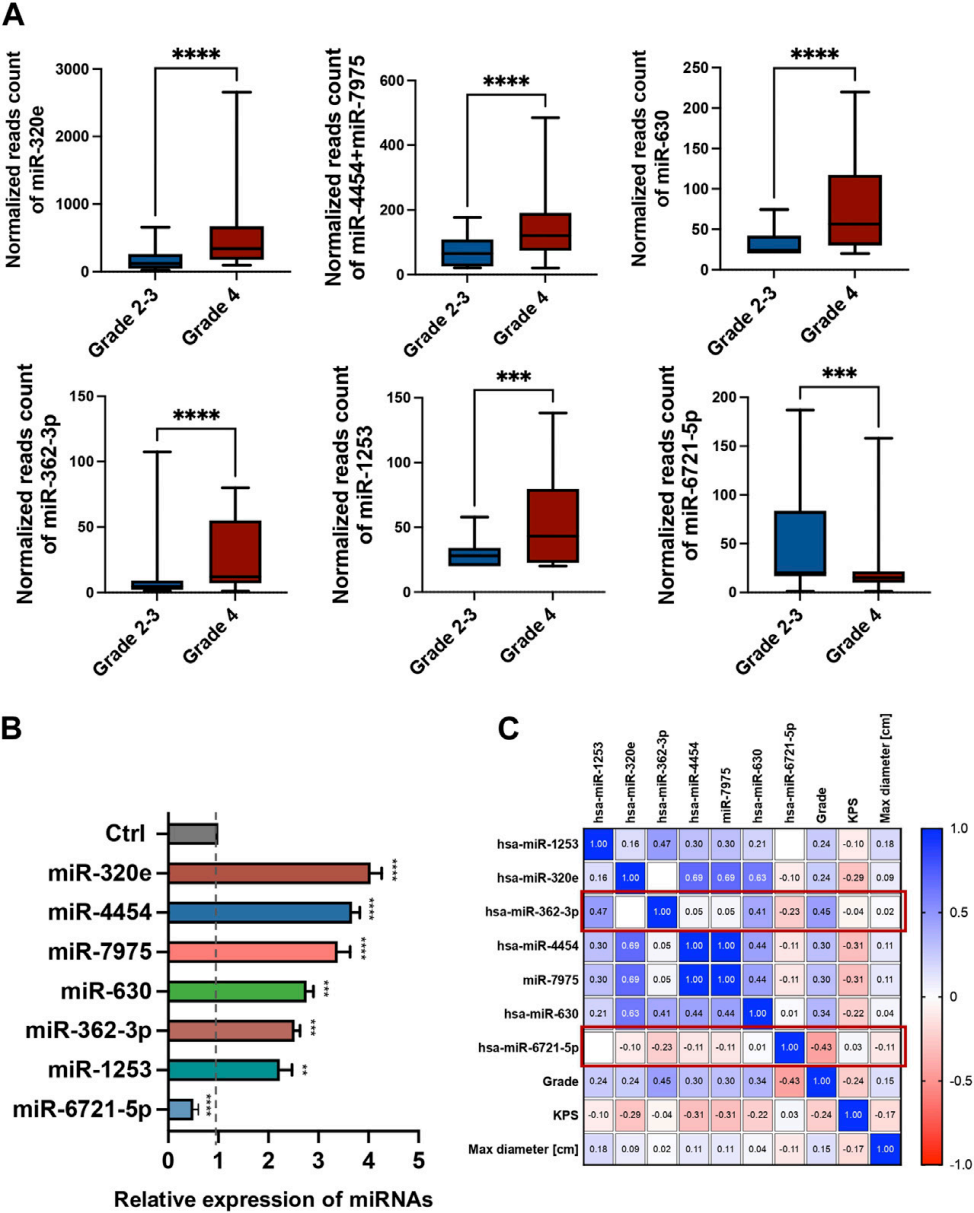
Differential miRNA expression in the serum of glioma patients **(A)** The bar plots show the expression levels of circulating miRNAs in serum in grade 4 glioma patients compared to grade 2–3 glioma patients from the NanoString platform. Differences in the expression levels of miRNAs between patients and controls were compared using the Mann-Whitney test. **(B)** RT-qPCR validation of seven miRNAs selected from the NanoString platform. Each bar represents the mean ratio of the differentially expressed (DE) miRNA expression and miR-103a-3p and miR-199-5p as reference miRNAs ± standard error of the mean (SEM). Asterisks indicate a significant difference compared to the control (****p < 0.0001, ***p < 0.001, **p < 0.01, *p < 0.05). **(C)** Pearson correlation results of the DE miRNAs and grade, Karnofsky Performance Score (KPS) and max diameter of tumour. The colour scale (blue to red) indicates correlation. The blue colour indicates a positive correlation, and red indicates the opposite.

The expression levels of 798 miRNAs were quantified in the study group consisted of patients qualified for surgical treatment of adult-type diffuse gliomas of different stages of development (according of WHO, 2021 classification), including 78 grade 4 gliomas and 24 grade 2 and 3. Altogether, seven unique miRNAs showed significant differences in counts between grade 4 gliomas and grade 2–3 gliomas serum samples with fold change (FC) > |1.5| and false discovery rates (FDRs) < 0.05 (Figure 1A). Table 2 summarises FCs and FDRs for all seven differentially expressed (DE) miRNAs. Five miRNAs were upregulated: miR-630, miR-362-3p, miR-320e, miR-4454+miR-7975 (combined because the mature sequence of miR-7975 differs from miR-4454 by only one base), whereas two miRNAs were downregulated: miR-1253 and miR-6721-5p. To validate the diagnostic usefulness of identified miRNAs, we performed RT-qPCR validation. All miRNAs were detectable by quantitative PCR, and the expression profiles were similar to the ones generated using the NanoString platform (Figure 1B). Next, we used Pearson’s correlation to determine a statistically significant relationship between miRNA expression levels and clinical features such as grade, Karnofsky Performance Score (KPS) and size of tumours (Figure 1C). The analysis indicated a positive correlation between miR-362-3p expression and a negative correlation between miR-6721-5p expression and gliomas’ grade with a coefficient of 0.45 and −0.43, respectively (*p*-value = 1.87 × 10^−6^ and *p*-value = 6.79 × 10^−6^). It means that the expression of miR-362-3p increased in the case of higher-grade gliomas, while the change in miR-6721-5p expression level was opposite to the change in the grade of gliomas.

**TABLE 2:** Overview of the DE miRNAs (−1.5 > FC > 1.5) in grade 4 patients concerning grades 2 and 3. The arrows symbolise a way of regulation (up or downregulation).

miRNA	FC	FDR
miR-630	1.98	<0.001
miR-362-3p	1.94	<0.001
miR-1253	−1.75	<0.001
miR-4454+miR-7975	2.04	<0.001
miR-320e	2.70	<0.001
miR-6721	−1.75	0.04

FC, fold change; FDR, false discovery rate.

### 3.2 Evaluation of diagnostic values of DE miRNAs

**FIGURE 2:**
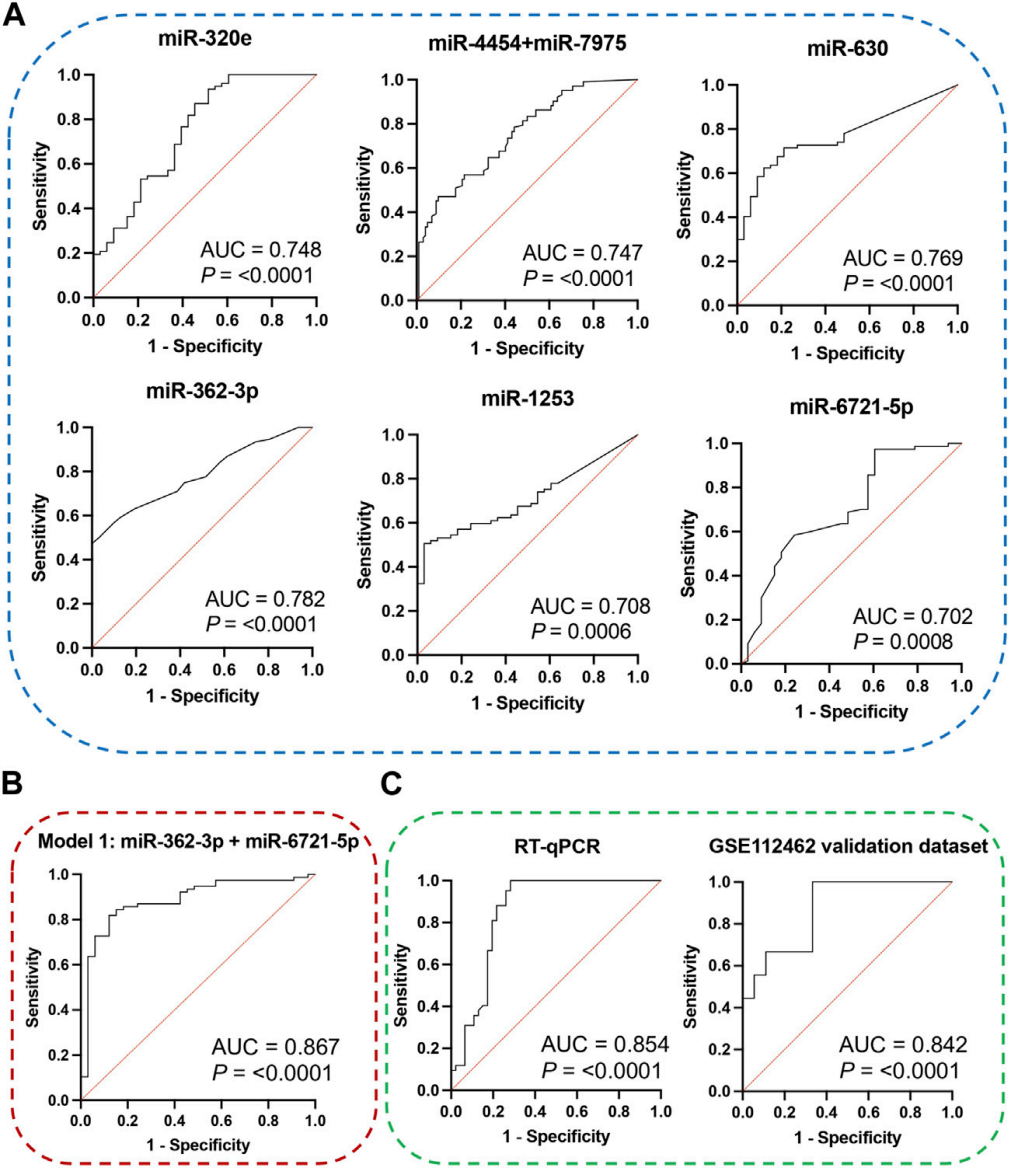
**(A)** ROC curves and AUC (Area Under the Curve) of DE miRNAs were obtained based on data on the expression level of miRNA molecules using the NanoString platform. **(B)** ROC curve and AUC for the diagnostic classification model based on data on the miR-362-3p and miR-6721-5p expression levels obtained using the NanoString platform. The graph consists of the AUC value, sensitivity and specificity corresponding to that point. **(C)** ROC curves and AUC for the diagnostic classification model were obtained on data using the RT-qPCR method and external public data, which is included in the Gene Expression Omnibus (GEO) database. The graphs contain the AUC value, sensitivity and specificity corresponding to that point.

The receiver operating characteristic (ROC) curve analysis was conducted to evaluate the diagnostic value of the identified miRNAs as biomarkers for binary classification (glioma 4 vs. glioma 2–3) (Figure 2A). The area under the ROC curve (AUC) for all miRNAs was higher than 0.700 (*p* < 0.0001). The highest AUC values were found for miR-362-3p, miR-630, and miR-320e (0.782, 0.769, and 0.748, respectively). Corresponding AUC values, confidence intervals (CI), sensitivity and specificity for cut-off points were calculated and are shown in Supplementary Table S3.

### 3.3 Identification of the best combination of miRNAs for grade 4 glioma detection

To select the combination of miRNAs with the strongest classification power for discrimination between grade 4 of adult-type diffuse glioma patients and lower grades, we applied the Information Gain (InfoGainAttributeEval) selection method in WEKA software to analyse obtained differentially expressed miRNA data. The Information Gain method with the Ranker Search method is based on the calculation of decreasing entropy by adding attributes, and Correlation-based feature selection prioritises uncorrelated features (Wainer and Cawley, 2021; Rafało, 2022). The three attributes by InfoGainAttributeEval Algorithms with the Ranker Search Method were miR-362-3p, miR-6721-5p, and miR-320e.

To assess the diagnostic values of the selected miR-362-3p, miR-6721-5p, and miR-320e, multivariate logistic regression was applied to develop the diagnostic models of the miRNAs under combination conditions. K-fold cross-validation (*K* = 10) was used to develop stable models. Based on the normalised NanoString data, four models were derived: model 1 was built on the expression of miR-362-3p and miR-6721-5p as independent variables; model 2 included miR-362-3p and miR-320e; model 3 was based on the expression of miR-6721-5p and miR-320e, while model 4 included all three variables, miR-362-3p, miR-320e, and miR-6721-5p (Table 3). To assess the specificity and sensitivity of the four models, ROC curves were drawn, as shown in Fig. XX. The AUC value for model 1 was 0.894, with the diagnostic sensitivity at 94.8% and the specificity at 69.6% (Figure 2B). For model 2, AUC was 0.851, sensitivity 83.1% and specificity 63.6 (Supplementary Figure S2A). For model 3, AUC was 0.831, sensitivity 89.6% and specificity 24.2% (Supplementary Figure S2B); for model 4, AUC was 0.862, sensitivity 92.8% and specificity 76.1% (Supplementary Figure S2C). The data suggested the best classification accuracy was achieved by model 1 (Table 4). In addition, the combination model showed a higher sensitivity and better specificity than individual miRNA.

**TABLE 3:** Summary of ROC parameters. AUC–area under curve, CI–confidence interval (95%), S–sensitivity and Sp–specificity.

DE miRNAs	AUC	Lower border CI (%)	Upper border CI (%)	S	Sp
miR-320e	0.748	0.64	0.85	68.8	63.6
miR-4454+7975	0.747	0.65	0.84	62.3	66.7
miR-630	0.769	0.68	0.85	72.7	72.7
miR-362-3p	0.782	0.69	0.86	71.0	61.3
miR-1253	0.708	0.61	0.80	62.3	63.6
miR-6721	0.702	0.59	0.81	59.7	69.7

**TABLE 4:** Summary of the basic parameters and standard quality measures of the models.

Name	Variables	TP rate	TN rate	Precision	MCC	AUC	Intercept	Coefficients
Model 1	x1 = miR-362-3p	0.948	0.696	0.879	0.687	0.894	0.7375	x1 = 0.082
	x2 = miR-6721-5p							x2 = −0.022
Model 2	x1 = miR-362-3p	0.831	0.636	0.842	0.463	0.851	−0.827	x1 = 0.016
	x2 = miR-320e							x2 = 0.001
Model 3	x1 = miR-6721-5p	0.896	0.242	0.734	0.180	0.831	0.764	x1 = 0.004
	x2 = miR-320e							x2 = 0.001
Model 4	x1 = miR-362-3p	0.928	0.761	0.780	0.695	0.862	2.348	x1 = 0.421
	x2 = miR-320e							x2 = 0.905
	x3 = miR-6721-5p							x3 = 0.998

T, true positive; FP, false positive; AU, the area under the curve.

### 3.4 Independent validation of the miR-362-3p and miR-6721-5p dual model

Next, RT-qPCR analysis was performed to further validate the value of circulating miR-362-3p and miR-6721-5p as prediction markers for different grated of gliomas. The expression levels of miR-362-3p and miR-6721-5p were normalised to the expression of reference miRNAs, miR-103-3p and miR-199-5p. A diagnostic classification model 5 was developed based on the normalised RT-qPCR data. As shown in Figure 2C; Table 5, model 5 provided very good discrimination between grade 4 glioma and lower grades, with the AUC value of 0.867, with a sensitivity of 82.9% and a specificity of 92.8%.

**TABLE 5:** Table of confusion in the set for Model 1.

		Actual state	
		miR-362-3p	
		miR-6721-5p	
		1	0
Prediction	1	71 (TP)	8 (FP)
	0	2 (FN)	21 (TN)

TN, true negatives; TP, true negatives; FN, false negatives; FP, false positives.

Next, further validation of the optimised miR-362-3p and miR-6721-5p signature was performed using an external dataset (GSE112462, NanoString human miRNA panel NS_H_MIR_V3A), which contains miRNA profiles of 28 glioma patients (18 patients with grade 2–3 and 10 patients with grade 4). We found that model 6, developed based on miR-362-3p and miR-6721-5p expression data in the GEO dataset, efficiently discriminated between grade 4 glioma patients and lower grade glioma individuals with AUC value of 0.842, sensitivity 66.7% and specificity 83.3% (Figure 2C; Table 6).

**TABLE 6:** Summary of the basic parameters and common quality measures of Model 5 for RT-qPCR data and Model 6 for external data from GEO database.

Name	Variables	TP rate	FP rate	Precision	MCC	AUC	Intercept	Coefficients
Model 5	x1 = miR-362-3p	0.829	0.928	0.765	0.676	0.854	0.5995	x1 = 0.250
	x2 = miR-6721-5p							x2 = 0.2240
Model 6	x1 = miR-362-3p	0.667	0.833	0.667	0.500	0.871	6.921	x1 = −0.954
	x2 = miR-6721-5p							x2 = −0.066

TP, true positive; FP, false positive; MCC, the Matthews correlation coefficient; AUC, the area under the curve.

### 3.5 Identification of hub genes using network-based DE miRNA target genes

**FIGURE 3:**
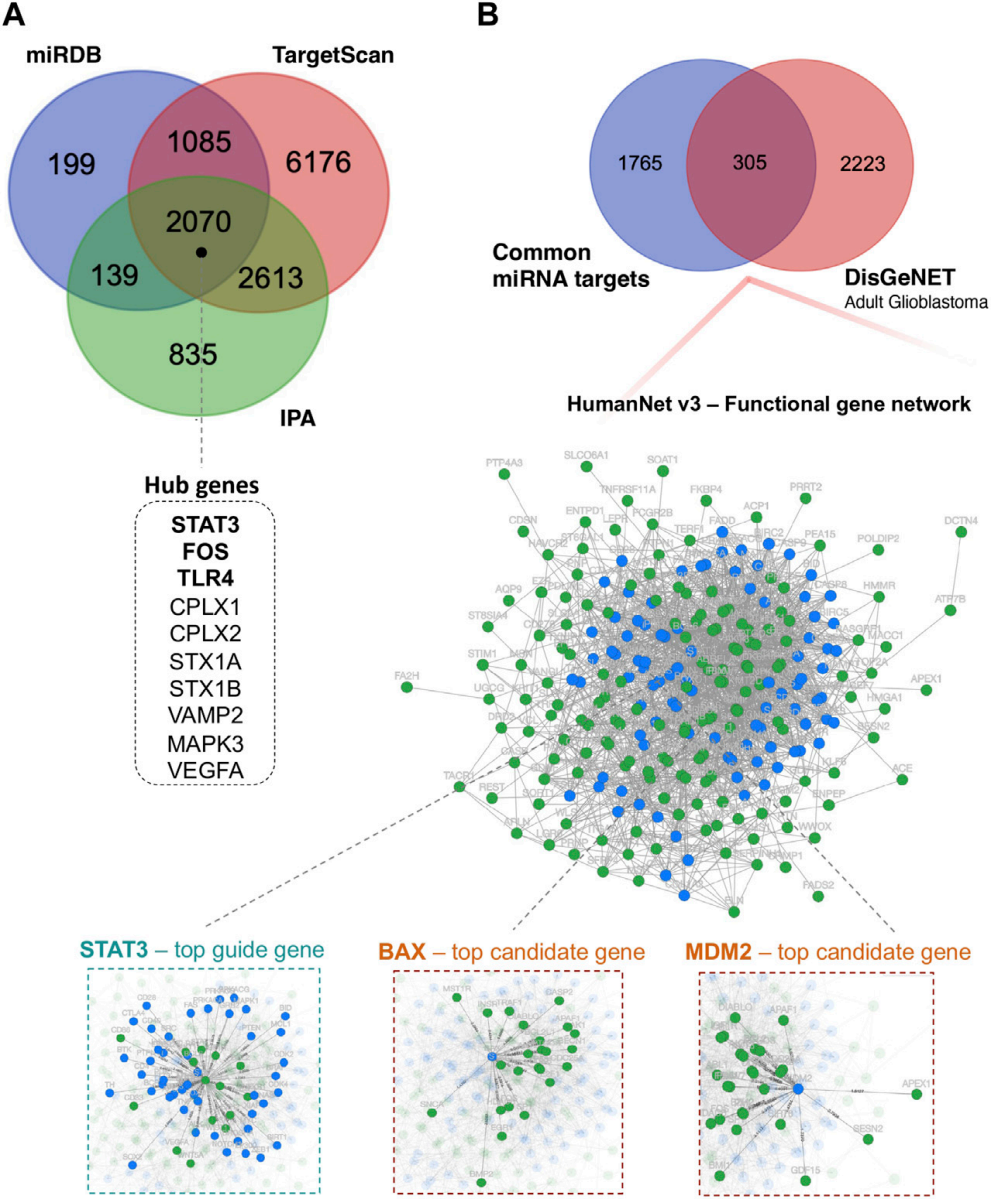
Functional annotation analysis of predicted miRNA-target genes. **(A)** Venn diagram showing the number of DE miRNA target genes shared between different databases: miRDB, TargetScan, and IPA; miRDB–microRNA Target Prediction Database; IPA–Ingenuity Pathway Analysis. **(B)** Venn diagram showing the overlap of 305 DE miRNA target genes and genes related to “adult glioblastoma” from the DisGeNET database. Additionally, integrated functional gene network analysis of 305 DE miRNA target genes with the HumanNet v3 platform. Network nodes represent guide (miRNA target genes–green) genes and candidate genes (blue). Edges represent their associations. Edges guide and edges between guide genes and candidate genes are presented; DisGeNET–a database of gene-disease associations.

To reveal the biological function of the DE miRNAs, first, we identified 5,657 putative DE miRNAs target genes using the Ingenuity Pathway Analysis (IPA) (Krämer et al., 2014), 3,493 target genes via mirDB (Chen and Wang, 2020) and 11,944 target genes via TagetScanHuman 8.0 (Agarwal et al., 2015) (Figure 3A). For further functional studies, we used 2,070 DE miRNA target genes that overlapped between the above databases. Using Cytoscape v.3.9.1 (Shannon et al., 2003), we constructed the protein-protein interaction (PPI) network, including 2,067 nodes and 12,250 edges, to identify the critical hub genes among the above miRNA target genes. The publicly available NetworkAnalyst platform (Zhou et al., 2019) was used to conduct “Zero order” interaction network analysis in a layout format using a force atlas to visualise the network, which included 514 nodes and 876 edges (Supplementary Figure S1). Hub genes in the network were ranked using eight topological analysis methods, including both local- and global-based algorithms from Cytoscape software’s cytoHubba plugin Pole (Chin et al., 2014). We found that *STAT3*, *FOS*, *TLR4*, *CPLX1*, *CPLX2*, *STX1A*, *STX1B*, *VAMP2*, *MAPK3*, and *VEGFA* scores ranked in the top (Figure 3A and Supplementary Table S2). The highly connected hub gene *STAT3* encodes signal transducer and activator of transcription. It is a multifunctional transcription factor involved in many biological functions ranging from initiating malignant transformation to tumour invasion, migration, metastasis and angiogenesis (Tolomeo and Cascio, 2021).

Next, we investigated whether and how these 2070 prioritised DE miRNA target genes could correlate with the stage of glioma. To this end, we used the HumanNet v3 platform (Kim et al., 2022). First, juxtaposed DE miRNAs target genes identified in our cohort with genes related to “adult glioblastoma” from the publicly available DisGeNET v7.0 database (Piñero et al., 2020). The 305 gene targets that overlapped between 2070 DE miRNAs target genes and 2,528 putative target genes related to “adult glioblastoma” were analysed in terms of known interactions using HumanNet v3 platform. Network analysis (Figure 3B) was performed based on HumanNet (functional gene network) which includes co-functional links (given by co-expression, co-essentiality, pathway database, protein domain profile associations, gene neighbourhood, and phylogenetic profile association) and protein-protein interactions. The top guide gene within this network was also *STAT3* (score = 42), and the other guide genes within the top scores included *TP53*, *MAPK3*, *SP1*, *PML*, *BCL2L1*, *E2F1*, *RELA*, *IGF1R*, and *CHEK1*. Within the obtained network, we also identified downstream candidate genes that could be functionally connected to the 305 input guide genes. The top scores comprised *STAT3* (score = 48.5), *BAX*, *MDM2*, *EGFR*, *PRKACA*, *PRKACB*, *SRC*, *MAPK1*, *MAPK8*, and *CTNNB1*.

**FIGURE 4:**
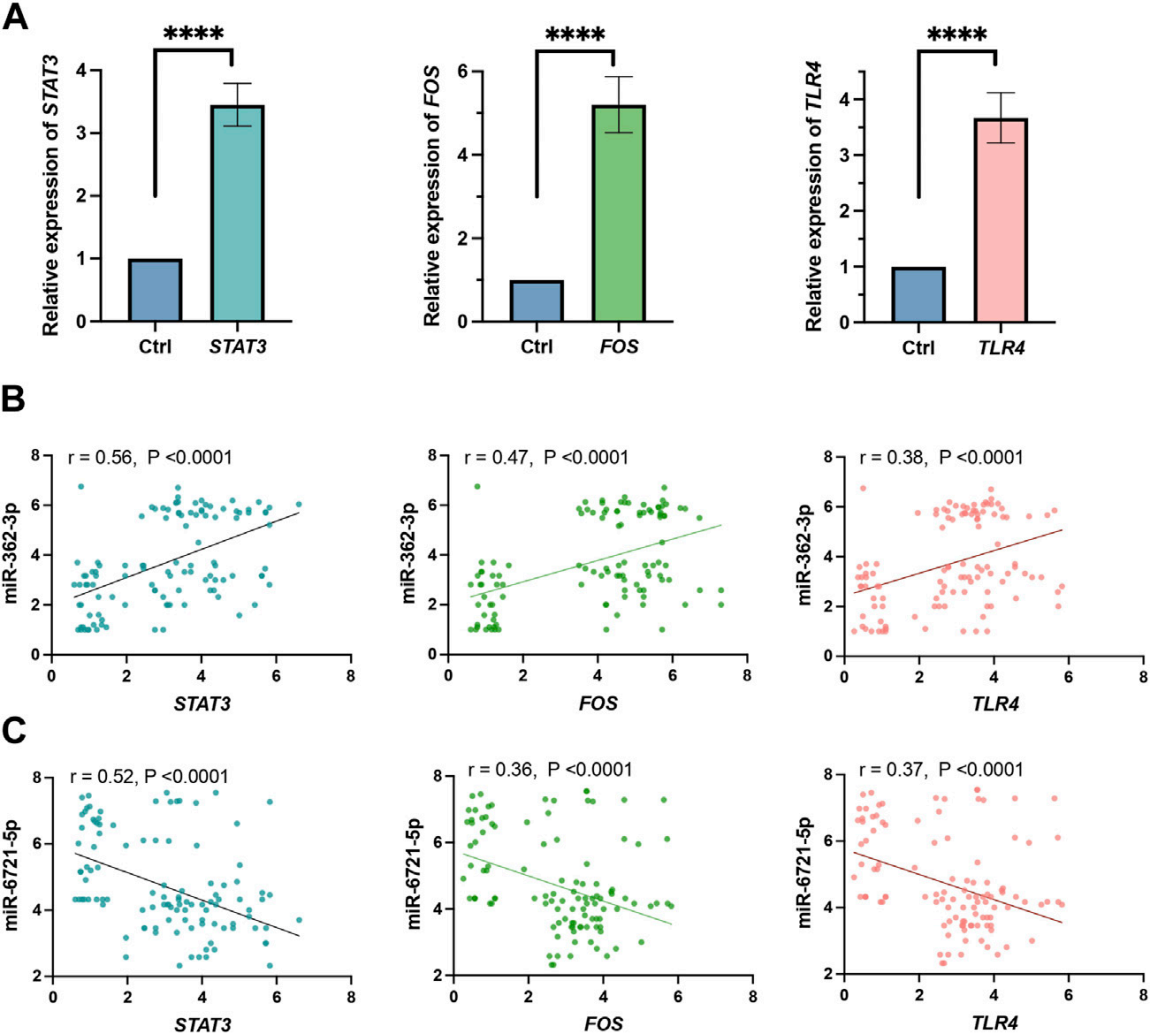
**(A)** Expression level obtained by RNA-seq of *STAT3*, *FOS* and *TLR4* genes. All gene expression levels between grade 4 glioma and grade 2–3 glioma patients differed significantly (****p < 0.0001). **(B)** Pearson correlation of miR-362-3p and *STAT3*, *FO*S and *TLR4* expression. **(C)** Pearson correlation of miR-6721-5p with *STAT3*, *FOS* and *TLR4* expression.

In addition, we evaluated the correlation between the expression of the top 3 hub genes, *STAT3*, *FOS* and *TLR4*, in the grade 4 glioma tissue and model 1 miRNAs, miR-362-3p and miR-6721-5p, in serum. Expression of all these genes increased in tumour samples (Figure 4A). The results, as shown in Figure 4B, demonstrate that the correlation between the expression of miR-362-3p and the expression of *STAT3*, *FOS*, and *TRL4* was moderately positive (0.56, 0.47 and 0.38, respectively) with a significant statistical value of *p* < 0.0001. Conversely, a moderate negative correlation was observed between miR-6721-5p and *STAT3*, *FOS* and *TRL4* (0.52, 0.36 and 0.37, respectively). The correlations were also statistically significant (*p* < 0.0001). It is worth emphasising that the highest value for the correlation coefficients was observed for both miRNAs and *STAT3*.

### 3.6 Functional enrichment analysis of the DE miRNA gene targets

To explore the potential biological functions and mechanisms of the DE miRNAs, 2070 DE miRNAs target genes were analysed by KEGG, WikiPathways and GO-BP pathway enrichment analyses using the enrichR tool (Kuleshov et al., 2016), as well as the canonical pathway enrichment analyses by the IPA (Krämer et al., 2014) Figure 5. The KEGG enrichment analysis indicated that DE miRNA target genes were mainly enriched in the JAK/STAT signalling pathway (FDR-corrected *p*-value = 5.34 × 10^−10^), HIF-1 signalling pathway (FDR-corrected *p*-value = 8.21 × 10^−10^), signalling pathways of pluripotency of stem cells (FDR-corrected *p*-value = 2.89 × 10^−9^), glioma-related pathway (FDR-corrected *p*-value = 5.94 × 10^−9^), and MAPK signalling pathway (FDR-corrected *p*-value = 2.15 × 10^−7^) (Supplementary Figure S3). It is noteworthy that the top 3 dysregulated pathways identified by the WikiPathways tool included the brain-derived neurotrophic factor (BDNF) signalling pathway (FDR-corrected *p*-value = 3.78 × 10^−9^), glial cell differentiation (FDR-corrected *p*-value = 4.16 × 10^−8^), and glioblastoma signalling pathway (FDR-corrected *p*-value = 6.92 × 10^−8^). BDNF regulates cell growth, differentiation, migration and apoptosis in the nervous system and high-grade gliomas (Xiong et al., 2013). The IPA analysis showed the involvement of the following canonical pathways: molecular mechanisms of cancer (FDR-corrected *p*-value = 7.45 × 10^−10^), glioma signalling (FDR-corrected *p*-value = 3.53 × 10^−8^), STAT3 signalling (FDR-corrected *p*-value = 1.63 × 10^−7^), glioblastoma invasiveness signalling (FDR-corrected *p*-value = 4.47 × 10^−7^), and synaptogenesis signalling pathway (FDR-corrected *p*-value = 2.29 × 10^−6^). The results of GO enrichment analysis showed that DE miRNA target genes were mainly related to the regulation of transcription and peptidyl-serine phosphorylation.

**FIGURE 5:**
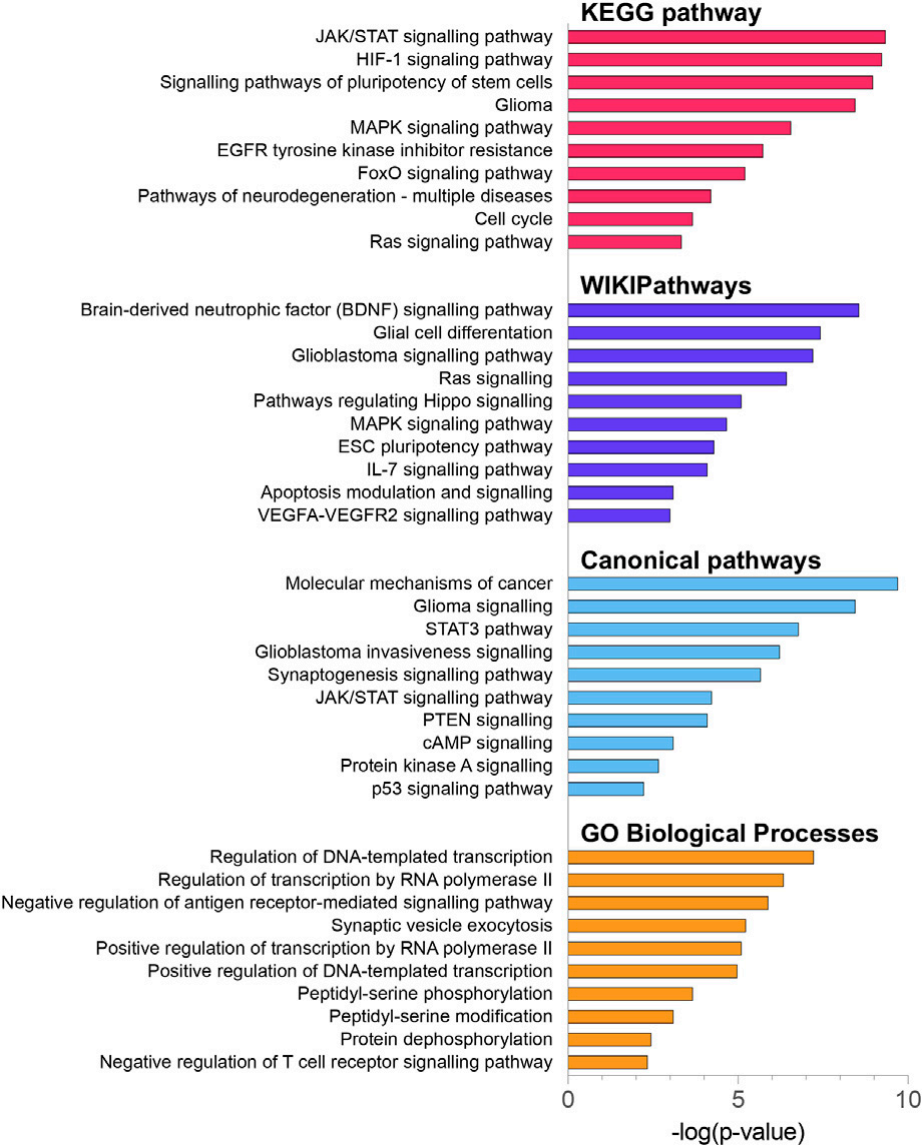
KEGG pathway, WikiPathway, canonical pathway and GO biological process enrichment analysis of 2070 DE miRNA target genes. All functional annotations were performed with the hypergeometric test and Bonferroni adjustment [corrected *p*-value (FDR) ≤ 0.05]; KEGG–Kyoto Encyclopedia of Genes and Genomes; GO–gene ontology.

### 3.7 Construction of weighted gene co-expression network and correlation with clinical traits

**FIGURE 6:**
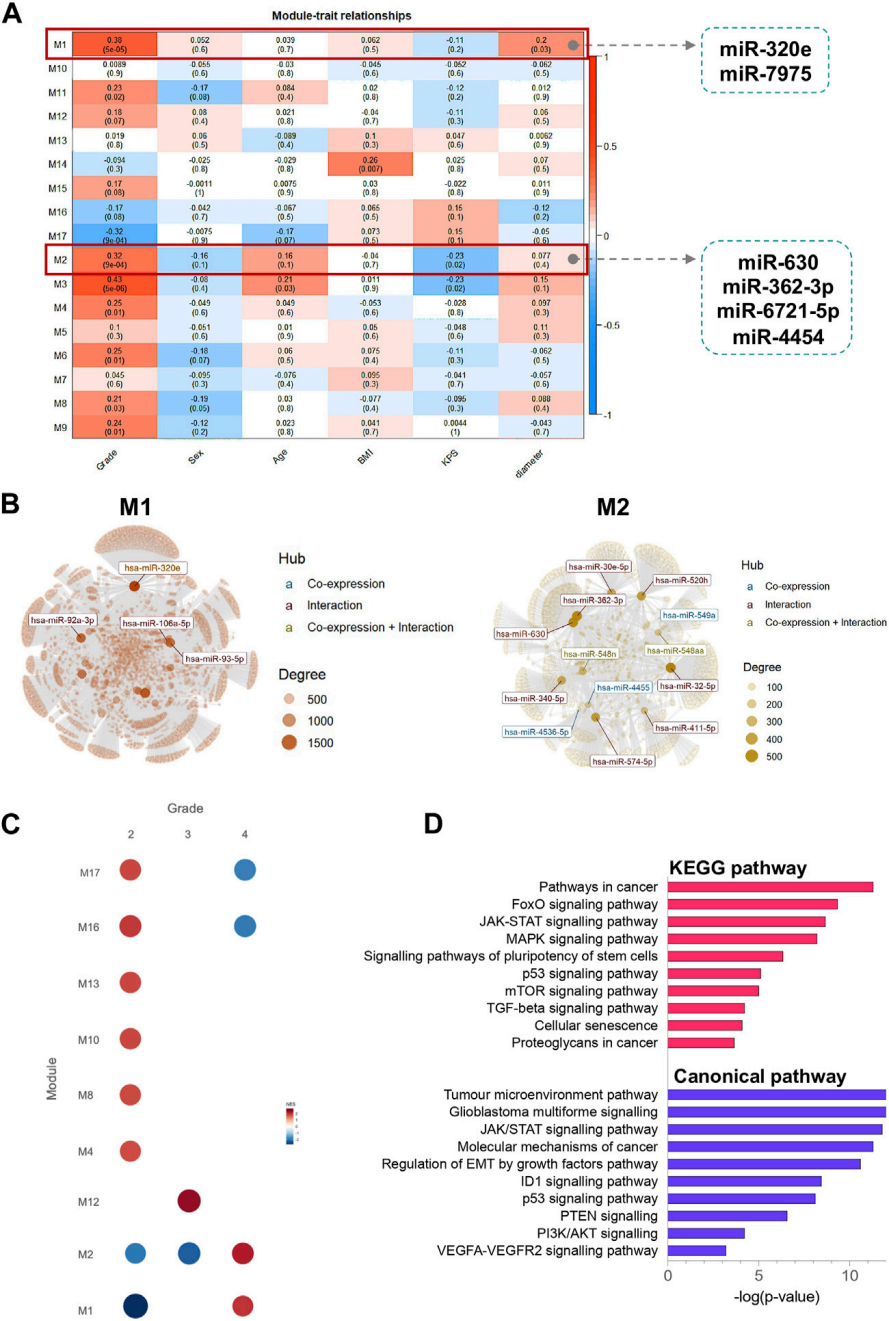
Identification of modules using WGCNA analysis associated with the clinical traits. **(A)** Heatmap of the correlation between module eigengenes and clinical characteristics; **(B)** miRNAs networks of modules M1 and M2. The top connected miRNAs (hubs) are labelled and coloured based on the interaction type (miRNA co-expression pattern–blue; miRNA-target gene interaction–red; miRNA co-expression pattern and miRNA-target gene interaction–yellow). The size of each node is proportional to its degree, as indicated in the plot legend. **(C)** Gene Set Enrichment Analyses showing the module activity of each grade of glioma. **(D)** KEGG pathway and canonical pathway enrichment analysis of miRNA target genes from modules M1 and M2. All functional annotations were performed with the hypergeometric test and Bonferroni adjustment [corrected *p*-value (FDR) ≤ 0.05]. KEGG–Kyoto Encyclopedia of Genes and Genomes.

To cluster DE miRNAs that are highly related to the clinical traits, we used the Weighted Gene Co-Expression Network (WGCNA) algorithm (Langfelder and Horvath, 2008) as a standard method to find cooperatively expressed mRNA or miRNA modules. The soft threshold value was set to 16, creating a scale-free system (Supplementary Figure S4). Modules were generated through dynamic tree cutting. After merging highly similar modules, we developed a total of 17 modules. Module M1 contained two DE miRNAs, miR-320e and miR-7975, whereas module M2 includes four DE miRNAs, miR-630, miR-362-3p, miR-6721-5p, and miR-4454. Additionally, we calculated the correlation between each module and clinical parameters. We identified that modules M1 and M2 were highly correlated with grade, KPS, and tumour diameter (Figure 6A). The modules M1 and M2 network graphs highlight significant miRNAs as network hubs, such as miR-320e in module M1, and miR-362-3p, miR-630, and miR-4454 in module M2 (Figure 6B). Gene set enrichment analysis revealed that modules M1 and M2 were positively associated with grade 4 gliomas (NES = 1.99 and NES = 2.16, respectively) and negatively associated with the lower grades. Finally, the results of the KEGG and canonical pathway enrichment analyses indicated that the miRNA target genes from modules M1 and M2 were mainly involved in the JAK-STAT signalling pathway (FDR-corrected *p*-value = 2.56 × 10^−13^), tumour microenvironment pathway (FDR-corrected *p*-value = 2.89 × 10^−13^), FoxO signalling pathway (FDR-corrected *p*-value = 1.08 × 10^−9^), glioblastoma multiforme signalling (FDR-corrected *p*-value = 3.11 × 10^−13^), and MAPK signalling pathway (FDR-corrected *p*-value = 4.26 × 10^−8^).

## 4 Discussion

MiRNAs are integrally involved in developing and progressing brain gliomas (Beylerli et al., 2022; Guo et al., 2022; Mafi et al., 2022). Since miRNAs contribute to the dysregulation of cell cycle control, cell proliferation, apoptosis, and other critical processes, their biomarker potential could improve brain tumour monitoring (Nikolova et al., 2024). Consequently, identifying unique serum miRNA signatures as circulating biomarkers could greatly benefit glioma management, allowing the avoidance of surgical biopsy for patients at high risk of mortality. It may provide valuable information regarding tumour status.

Our study strength lies in its innovative approach, which enables the identification of a specific serum-derived miRNA signature in glioma patients. Furthermore, we demonstrated that a two-miRNA biomarker signature can successfully differentiate between grade 4 and lower-grade gliomas, improving diagnosis and tailoring treatment options. To our knowledge, no currently known miRNAs are specific for WHO CNS5 grade 4 gliomas. Thus far, most research has relied on the WHO CNS4 (2016) classification and frequently utilised qPCR to analyse a limited number of miRNA candidates as biomarkers. Following the 2016 WHO CNS4 guidelines, LGG (grades I and II) IDHmut samples are classified as oligodendroglioma in the presence of 1p/19q codeletion, while they are astrocytoma in the absence of 1p/19q codeletion. LGG samples with IDHwt can be classified as other glioma types. GBM can be classified as IDHmut or IDHwt. In contrast, grade 4 adult gliomas, according to the WHO CNS5 classification, are defined as IDH-mutant astrocytoma and wild-type IDH glioblastomas (WHO, 2021). These tumours histologically manifest necrosis and/or microvascular proliferation. Additionally, WHO CNS5 grade 4 gliomas are characterised by the typical brain-blood barrier (BBB) breakdown. This dysfunctional barrier enables the transport of miRNAs to body fluids, making them promising biomarkers for gliomas.

In this study, we assessed miRNA signatures in preoperative serum samples obtained from 78 patients with grades 4 and 24 with grades 2–3 gliomas, which were classified according to the latest WHO CNS5 standards. Our study based on the rigorous methodological design. To minimize potential inaccuracies due to sample processing, our biobank has worked with the strictest standard operating procedures. We also used a workflow with highly validated procedures for miRNA expression analysis. In the discovery phase, we used the nCounter^®^ platform, to detect miRNAs without relying on reverse transcription and amplification, minimizing potential biases in experimental outcomes. It is worth noting that comparing PCR-based methods and high-multiplex approaches such as RNA-seq and microarray, NanoString nCounter is more accurate and scalable and its results are highly reproducible (Hong et al., 2021). Our findings indicate that the expression levels of seven circulating miRNAs (miR-320e, miR-4454, miR-7975, miR-630, miR-362-3p, miR-1253, and miR-6721-5p) were significantly altered in grade 4 gliomas before surgery, as compared to the lower grade cases. Notably, most miRNAs in this group have been previously linked to cancer and/or glioma. For instance, Pan et al. demonstrated a significant reduction of miR-320e expression in glioma tissue compared to healthy tissue. MiR-320e has been identified as a potential therapeutic target up-regulating the PBX2/Raf-1/MAPK axis in glioma (Pan et al., 2017). Similarly, miR-630 expression was downregulated in CNS4 WHO glioblastoma (GBM) compared to non-neoplastic white matter samples (Junior et al., 2020). Furthermore, overexpression of miR-630 in the U87 GBM cell line reduced cell proliferation and invasion. Similarly, Shi et al. demonstrated that miR-362-3p expression was also reduced in GBM tissue, which correlated with poor prognosis in GBM patients. MiR-362 targets mitogen-activated protein kinase 1 (MAPK), contributing to tumour development (Shi et al., 2020). Our study shows that the serum of grade 4 patients contained decreased levels of three miRNAs, miR-320e, miR-630, and miR-362-3p. As shown above, all of them can be considered tumour suppressors. It has been recently reported that tumour-suppressive miRNAs are significantly downregulated in glioblastoma tissue (Rupaimoole et al., 2016; Chhatriya et al., 2019). The release mechanisms of miRNAs from cancer cells and the process of crossing the blood-brain barrier to enter systemic circulation remain unclear. However, studies have identified that extracellular miRNAs present in the blood are bound to Ago2 protein or encapsulated in exosome (Skog et al., 2008; Shea et al., 2016). For instance, Qi et al. discovered that oncosuppressors, including miR-1298-5p, miR-122-5p and miR-204-5p, are absolutely sorted into exosomes, leading to the downregulation of their pool in tumour tissue (Qi et al., 2022). Conversely, the oncomiR miR-9-5p remained trapped within the tumour cells (Bandini et al., 2020).

Based on the miRNA’s expression profile in the serum of WHO CNS5 grade 4 patients, we proposed a classification model to improve the diagnosis of the highest grade of adult-type diffuse gliomas. We identified two-miRNA biomarker signatures, miR-362-3p and miR-6721-5p, effectively differentiating between WHO CNS5 grade 4 and lower-grade gliomas with an AUC of 0.894, 94.8% sensitivity and 69.6% specificity. To date, numerous studies have explored the potential of miRNAs as serum biomarkers for gliomas. However, most analyses have relied on the WHO CNS4 2016 classification and focused on distinguishing between grade III-IV glioma patients and those with grade I-II gliomas, differentiating glioma and non-cancer controls, or developing biomarkers specifically for glioblastoma multiforme (GBM). To the best of our knowledge, we show for the first time that a diagnostic model based on combined expression miR-362-3p and miR-6721-5p has the potential to differentiate grade 4 adult-diffuse gliomas from lower grade classified based on WHO CNS5 standards.

Furthermore, analysis of miRNA expression concerning clinicopathological factors like grade of tumour, KPS and tumour size confirmed that the level of both miR-362-3p and miR-6721-5p miRNAs exhibited a correlation with glioma grading. Our studies are following the latest observations showing that miR-362-3p, along with miR-3651 and let-71-3p, are the most significant contributors to stage prediction across eight types of cancer, including bladder carcinoma, breast invasive cancer, oesophageal carcinoma, kidney renal clear cell carcinoma, lung adenocarcinoma, stomach adenocarcinoma, and uveal melanoma (Yerukala Sathipati et al., 2023). Similarly, Tito et al. also demonstrated that miR-362-3p has diagnostic potential as a part of a signature panel comprising miR-193-3p, miR-572, miR-28-5, and miR-378, with an AUC of 0.801 in stage I of clear cell renal cell carcinoma (Tito et al., 2021). Additionally, miR-362-3p has been detected in the plasma of patients with colorectal cancer (CRC). Its levels were significantly lower in CRC patients compared to healthy individuals or patients with benign colorectal conditions (Mehrgou et al., 2021). miR-362-3p has also been significantly altered in the serum for lung cancer patients. Its levels are correlated with the tumour stage and might be associated with lung cancer development and metastasis (Wani et al., 2022). These studies suggest that miR-362-3p has the potential to be utilised as a circulating biomarker for various cancers.

Our analysis of predicted differentially expressed miRNA target genes showed gene enrichment in critical cancer pathways linked to tumorigenesis, including the RAS, STAT3, FoxO, MAPK, PI3K-Akt signalling pathways, as well as glioblastoma invasiveness signalling. These pathways are involved in the regulation of proliferation, differentiation, survival, and angiogenesis. RAS protein signalling is essential for WHO CNS4 glioblastoma (GBM) tumorigenesis (Pearson and Regad, 2017). RAS activates RAF/MEK/MAPK and PI3K/AKT cascades to promote cell proliferation, survival and uncontrolled growth of GBM (Fonseca et al., 2008; Mao et al., 2012).

In addition to these pathways, we identified the top 10 hub genes by network-based analysis, including *STAT3*, *FOS*, *TLR4*, *CPLX1*, *CPLX2*, *STX1B*, *VAMP1*, *MAPK3*, and *VEGFA*. Leveraging the HumanNet-FN tool, we also identified top guide and candidate genes within the created networks based on overlapping putative targets mRNA identified for the serum miRNA profile and genes related to adult glioblastoma by the DisGenNET databases. *STAT3* was recognised as the top guide gene. The abnormal activation of STAT3 promotes tumour proliferation, angiogenesis, and immune escape (Fu et al., 2023). Additionally, STAT3 is necessary for the proliferation and maintenance of pluripotency of GBM stem cells (Sherry et al., 2009). Genes encoding SNARE proteins, including syntaxin, Stx1, Stx2, and Vamp2, were also identified as hub putative DE miRNA target genes. These proteins are required for calcium-dependent exocytosis and neurotransmitter release within the nervous system (Jahn and Fasshauer, 2012). Given that SNARE proteins are involved in neuronal migration and that GBM is an extremely invasive tumour, it has been shown that Stx1 inactivation significantly reduced the growth and progression of GBM *in vivo* (Ulloa et al., 2015).

In conclusion, we have identified seven differentially expressed serum-derived miRNAs in WHO grade 4 gliomas and presented their functional analysis. Moreover, we developed a diagnostic model combining two serum-circulating miR-362-3p and miR-6721-5p expressions, which could be used as a non-invasive tool for grading gliomas in clinical applications.

Despite the novelty aspect, our study also has some limitations. Firstly, the sample size is limited; thus, results should be considered preliminary. Secondly, despite the effort to balance cancer samples while building the grade 4 glioma diagnosis model, there were still some imbalances in the size of groups of different glioma stages while constructing the model, resulting in over-representation of grade 4 glioma over grade 2–3. Therefore, further validation by multiple-centre and large-scale investigations using larger patient cohorts will be required to help strengthen the value of the two-miRNA model for clinical application.

In summary, this work presents a candidate non-invasive biomarker for a grade prediction of adult-type diffuse glioma. We verified that the increased expression levels of circulating miR-362-3p and reduced circulating miR-6721-5p displayed a high diagnostic value to differentiate grade 4 glioma patients from lower grade cases (grade 2–3). Our study demonstrates the efficacy of the novel approach for grade classification of glioma. The implementation of this model could offer complementary support to the preoperative diagnostic. Furthermore, our model could complement neuroradiology to support clinical decision-making and could be used in routine screening.

## Data Availability

The datasets presented in this study can be found in online repositories. The names of the repository/repositories and accession number(s) can be found below: https://www.ncbi.nlm.nih.gov/geo/, GSE247312.
