# Supplementary material for: Circulating serum miR-362-3p and miR-6721-5p as potential biomarkers for classification patients with adult-type diffuse glioma
Source: Front Mol Biosci. 2024 Feb 22;11:1368372. doi: 10.3389/fmolb.2024.1368372 (PMC10918470; doi:10.3389/fmolb.2024.1368372)
Supplement: Supplementary file 1 [file Table1.docx]

Supplementary Material

Circulating serum miR-362-3p and miR-6721-5p as potential biomarkers for classifying patients with adult-type diffuse glioma

Magdalena Niemira^1*^, Agnieszka Bielska^1^, Karolina Chwialkowska^2^, Justyna Raczkowska^1^, Anna Skwarska^3^, Anna Erol^1^, Anna Zeller^1^, Gabriela Sokolowska^1^, Damian Toczydlowski^1^, Iwona Sidorkiewicz^1^, Zenon Mariak^4^, Joanna Reszec^5^, Tomasz Lyson^4^, Marcin Moniuszko^6^, Adam Kretowski^1,7^

*** Correspondence:** Corresponding Author: [magdalena.niemira@umb.edu.pl](mailto:magdalena.niemira@umb.edu.pl)

**Supplementary Table S1.** List of Assay ID (Qiagen, Germany) and DE miRNAs sequences.

| miRNA | Assay ID | Sequence (5’ to 3’) |
| --- | --- | --- |
| miR-630 | YP00204392 | AGUAUUCUGUACCAGGGAAGGU |
| miR-362-3p | YP00205612 | AACACACCUAUUCAAGGAUUCA |
| miR-1253 | YP00204525 | AGAGAAGAAGAUCAGCCUGCA |
| miR-4454 | YP02114119 | GGAUCCGAGUCACGGCACCA |
| miR-7975 | YP02107687 | AUCCUAGUCACGGCACCA |
| miR-320e | YP02103235 | AAAGCUGGGUUGAGAAGG |
| miR-6721-5p | YP02110957 | UGGGCAGGGGCUUAUUGUAGGAG |
| miR-103-3p | YP00204063 | AGCAGCAUUGUACAGGGCUAUGA |
| miR-199b-5p | YP00204152 | CCCAGUGUUUAGACUAUCUGUUC |

**Supplementary Table S2.** Hub genes for miRNA targets ranked by different CytoHubba methods. MCC, the maximal clique centrality; MNC, the maximum neighbourhood component.

| **Category** | **Rank methods in CytoHubba** | | | | | | | |
| --- | --- | --- | --- | --- | --- | --- | --- | --- |
|  | **MCC** | **MNC** | **Degree** | **BottleNeck** | **Betweenness** | **Closeness** | **Stress** | **Radiality** |
| 1 | **STAT3** | **PRKACB** | **PRKACB** | **PRKACB** | **PRKACB** | **PRKACB** | **PRKACB** | **PRKACB** |
| 2 | **PRKACB** | **TLR4** | **TLR4** | **STAT3** | **STAT3** | **TLR4** | **STAT3** | **TLR4** |
| 3 | **CPLX2** | **CPLX2** | **STAT3** | **TLR4** | GRIN1 | **STAT3** | DLG4 | **STAT3** |
| 4 | **CPLX1** | **STAT3** | DLG4 | **VEGFA** | FOS | GRIN1 | **TLR4** | HTT |
| 5 | **TLR4** | **FOS** | **VEGFA** | ABL1 | **VEGFA** | **CPLX1** | **VEGFA** | **VEGFA** |
| 6 | **STX1A** | **VEGFA** | **CPLX2** | DLG4 | **STX1B** | **CPLX2** | **CPLX1** | **MAPK3** |
| 7 | **VAMP1** | **STX1B** | **CPLX1** | **MAPK3** | **VAMP1** | FOS | **MAPK3** | **VAMP1** |
| 8 | **STX1B** | FOS | FOS | **CPLX1** | **MAPK3** | **MAPK3** | GRIN1 | **STX1B** |
| 9 | VAMP7 | **VEGFA** | **MAPK3** | **CPLX2** | CANX | **VEGFA** | **STX1B** | **CPLX2** |
| 10 | STAT1 | **MAPK3** | **VAMP1** | MAPT | MAPT | **STX1B** | CANX | SNCA |


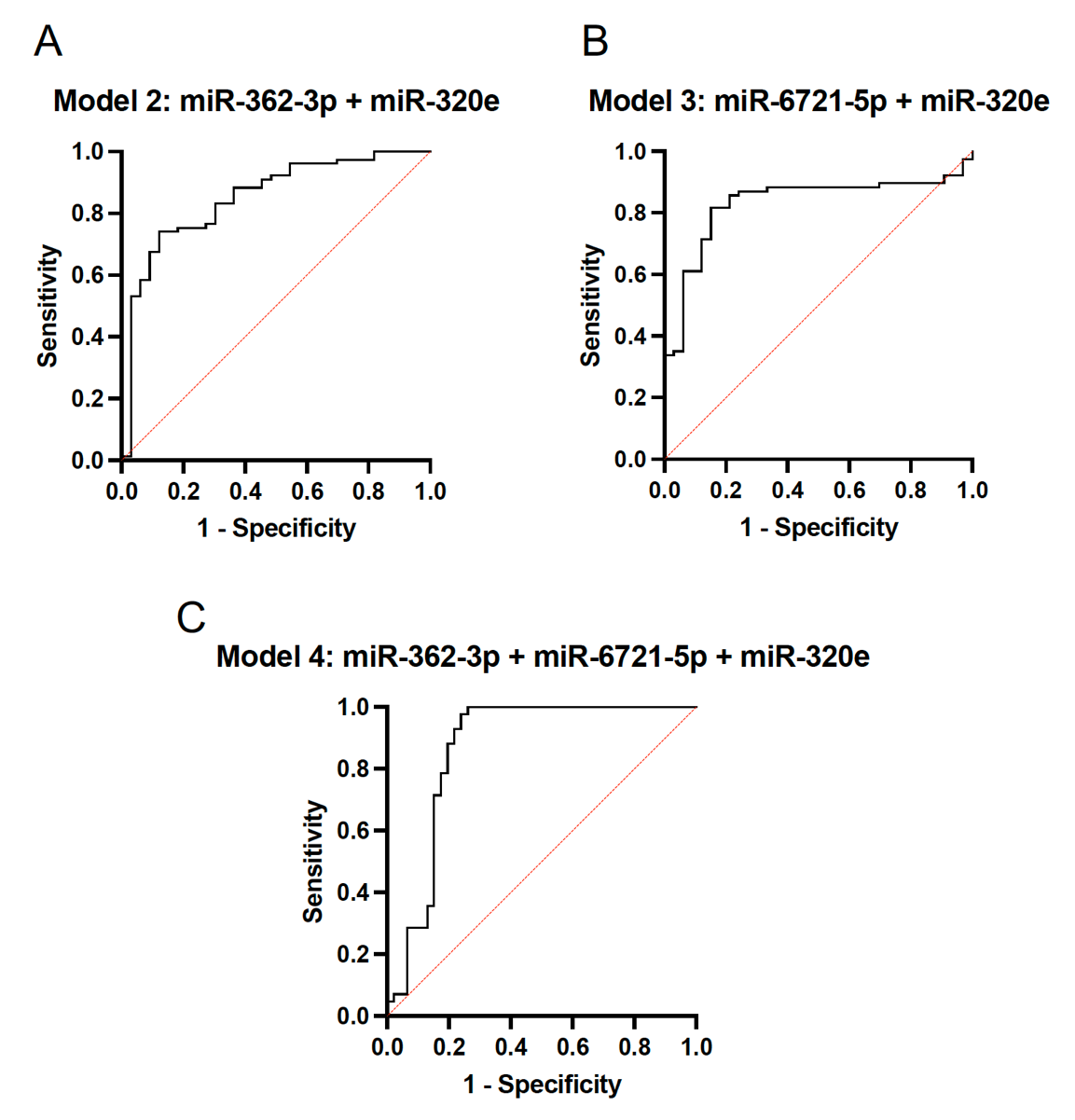


**Supplementary Figure 1.** (A) ROC curve and AUC for the diagnostic classification model based on data on the miR-362-3p and miR-320e expression levels obtained using the NanoString platform. The graph consists of the AUC value, sensitivity and specificity corresponding to that point. (B) ROC curve and AUC for the diagnostic classification model based on data on the miR-6721-5p and miR-320e expression levels obtained using the NanoString platform. The graph consists of the AUC value, sensitivity and specificity corresponding to that point. (C) ROC curve and AUC for the diagnostic classification model based on data on the miR-362-3p, miR-6721-5p and miR-320e expression levels obtained using the NanoString platform. The graph consists of the AUC value, sensitivity and specificity corresponding to that point.

**
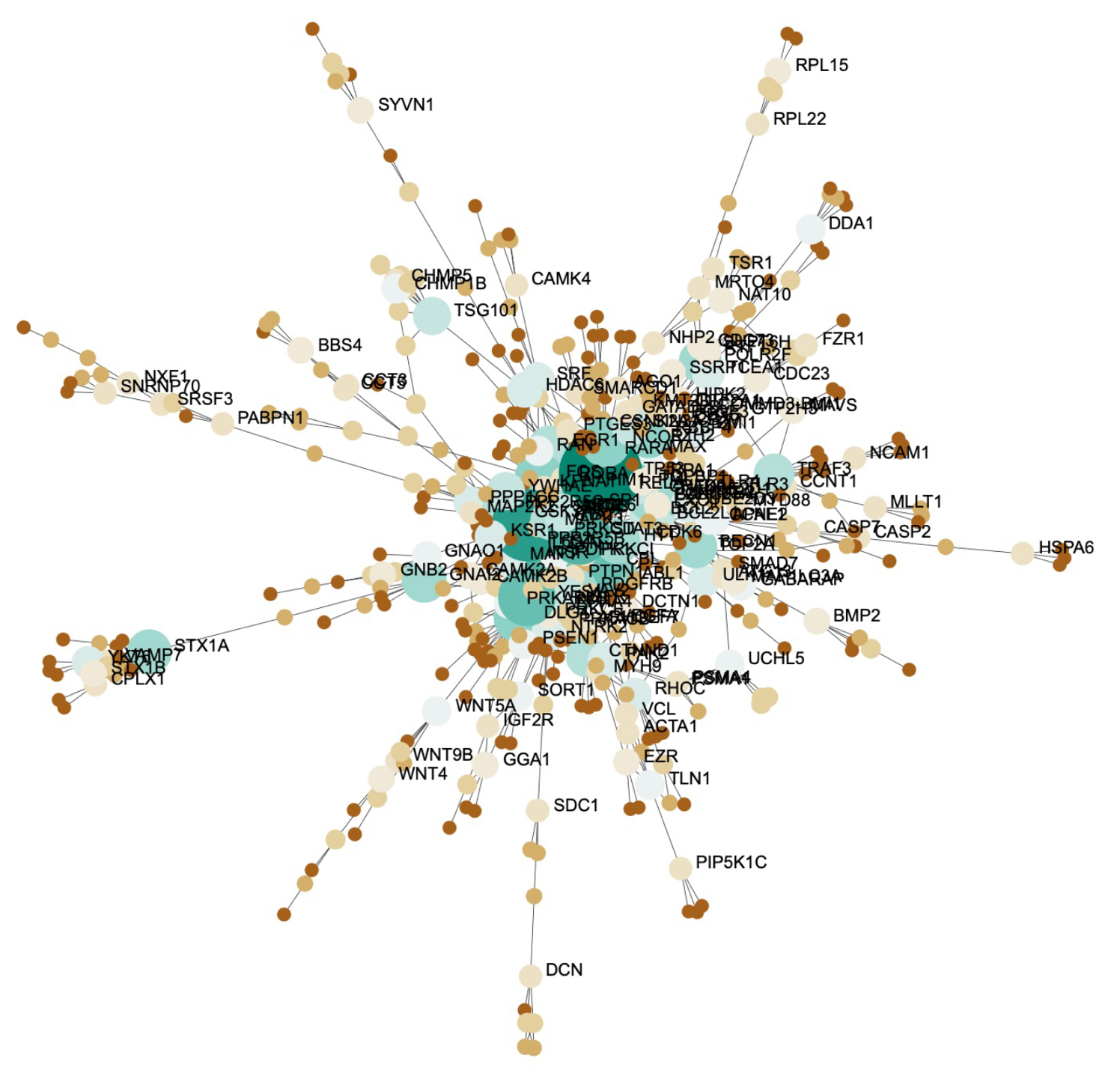
**

**Supplementary Figure S2.** Zero-order protein-protein interaction (PPI) network of DE miRNAs target genes. Data was drawn using the NetworkAnalyst 3.0 platform. Node size reflects the number of protein-protein interactions, with larger nodes representing a greater number of annotated interactions.

**
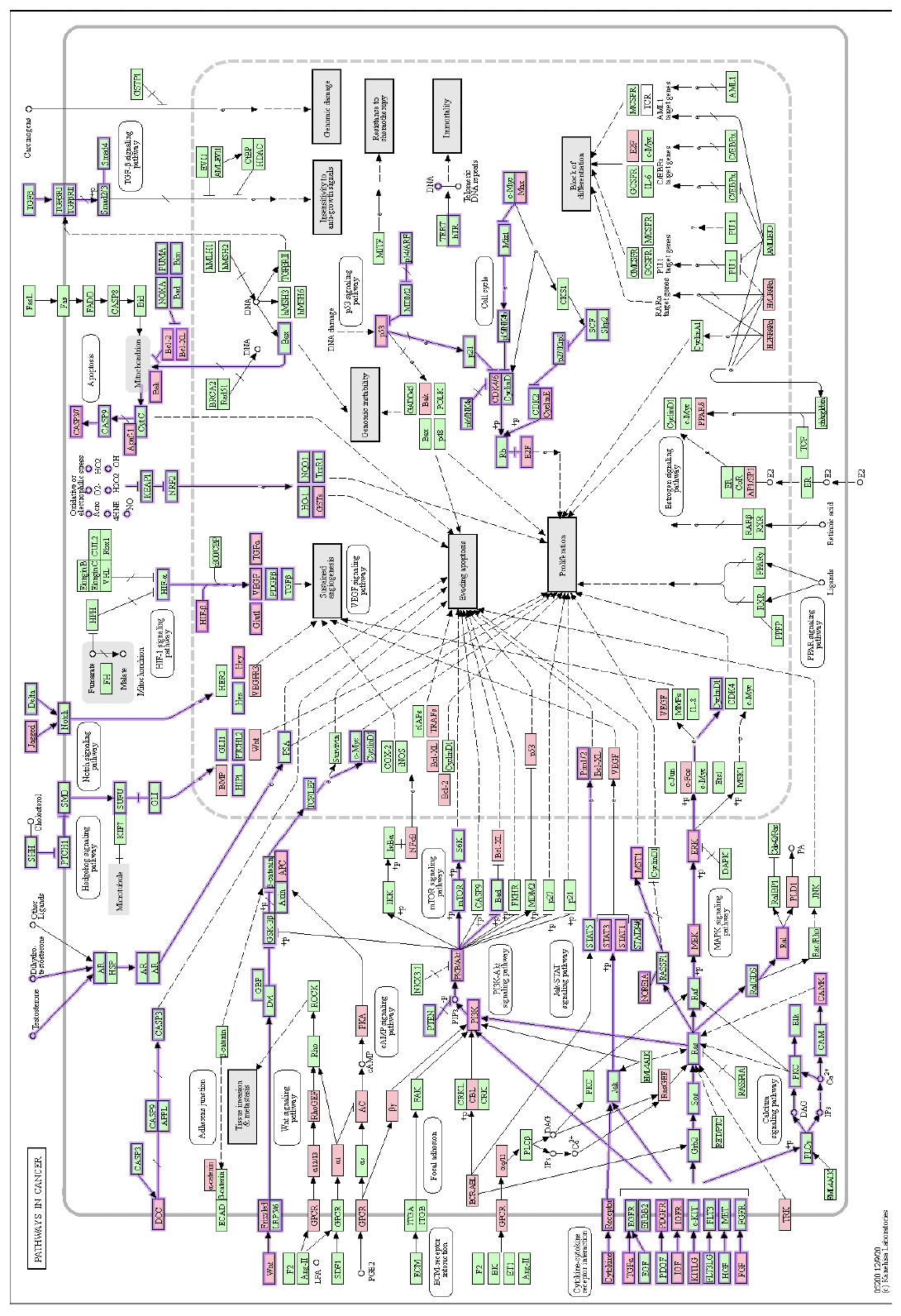
**

**Supplementary Figure S3.** KEGG pathway map for “Pathways in cancer” displaying DE miRNAs target genes. The genes that are red-coded are DE miRNAs targets. The purple line indicates the individual pathways.


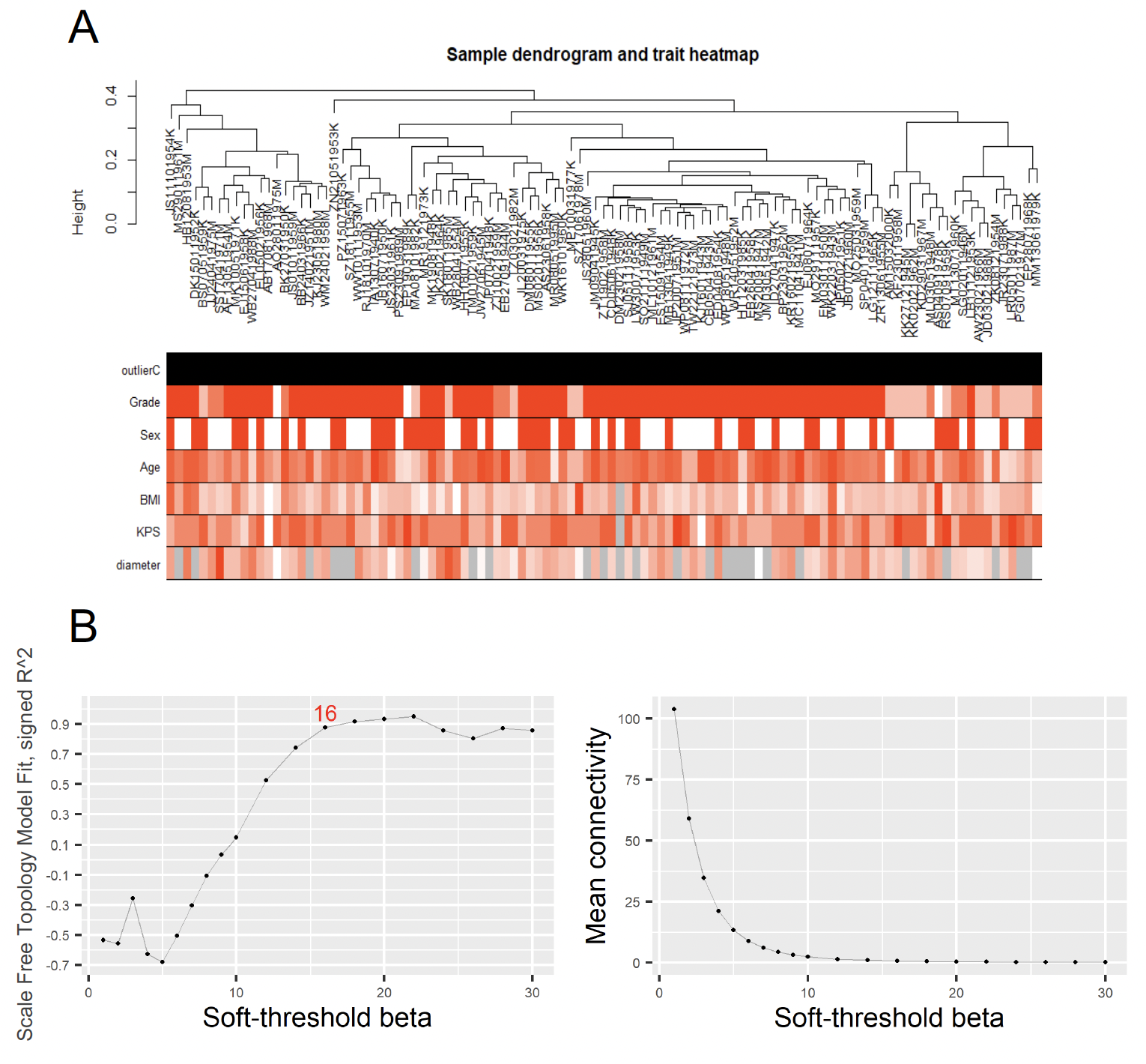


**Supplementary Figure S4.** Weighted Gene Co-expression Network Analysis (WGCNA). (A) Cluster analysis of samples of glioma patients to detect outliers (white-to-red linear gradient colour associated with a corresponding clinical variable, grey when missing data); (B) Determination of soft-thresholding power in weighted gene co-expression network analysis (WGCNA).
